# Arachnoid Cyst: An Asymptomatic Exuberance

**DOI:** 10.7759/cureus.31782

**Published:** 2022-11-22

**Authors:** André Santos, Ana Filipa Viegas, Lenea M Porto, Ana Gomes, Edite Nascimento

**Affiliations:** 1 Internal Medicine, Centro Hospitalar Tondela-Viseu, Viseu, PRT; 2 Stroke Unit, Centro Hospitalar Tondela-Viseu, Viseu, PRT

**Keywords:** stroke mimics, focal neurologic deficit, intracranial lesions, peritoneal shunt, neurosurgery, arachnoid cyst

## Abstract

A 66-year-old man presented to the emergency department with sudden onset of dysarthria, left central facial palsy, and left hemihypesthesia involving the tongue. The patient was hemodynamically stable (blood pressure of 153/84 mmHg and heart rate of 80 bpm) and normoglycemic, without a history of trauma or toxic exposure. Assuming an acute stroke, the patient immediately underwent a brain CT scan that revealed a large left-sided fronto-parieto-temporal arachnoid cyst, with approximately 9.5 x 5.1 cm of maximum diameters (anteroposterior and transversal), compressing the brain parenchyma and the ventricular system, with a right deviation of the median structures by about 5 mm. The patient had a complete spontaneous resolution of the initial symptoms while in the emergency department. He declined admission to the ward for observation and further investigation, choosing to be discharged against medical advice. Lately, the patient represented to the ED with a new episode, this time with worsening symptoms, and consented to a cystoperitoneal shunt insertion. The procedure was well tolerated, and the patient has been asymptomatic since surgery.

## Introduction

Arachnoid cysts consist of an intracranial cerebrospinal fluid (CSF) collection covered by an arachnoid similar membrane between the dura mater and pia mater. Usually, with a benign evolution, they represent approximately 1% of the intracranial expansive lesions. The signs and symptoms vary according to their size and location. Arachnoid cysts may present as asymptomatic incidental neuroimaging findings, generally small cysts, or provoking severe headaches, seizures, hydrocephaly, intracranial hypertension, cranial nerves palsies, vertigo, proptosis, hemiparesis, and mental status changes due to mass effect caused by larger cysts [1,2]. Arachnoid cysts may have a fast evolution. They may present spontaneous growth, size reduction, or even total disappearance [3].

## Case presentation

**Figure 1:**
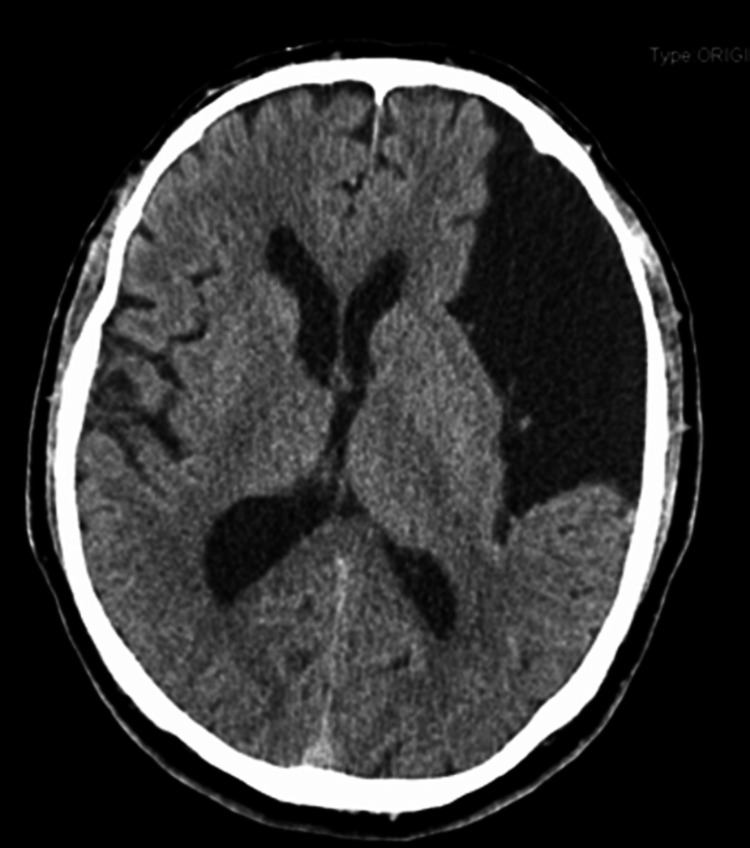
Brain computed tomography Brain computed tomography in axial view showing a large left fronto-parieto-temporal extra-axial fluid collection measuring 9.5 x 5.1 cm of maximum diameters creating a significant parenchymal and ventricular compression with subfalcine herniation (midline shift). It is also possible to see a 5 mm deviation of midline structures.

**Figure 2:**
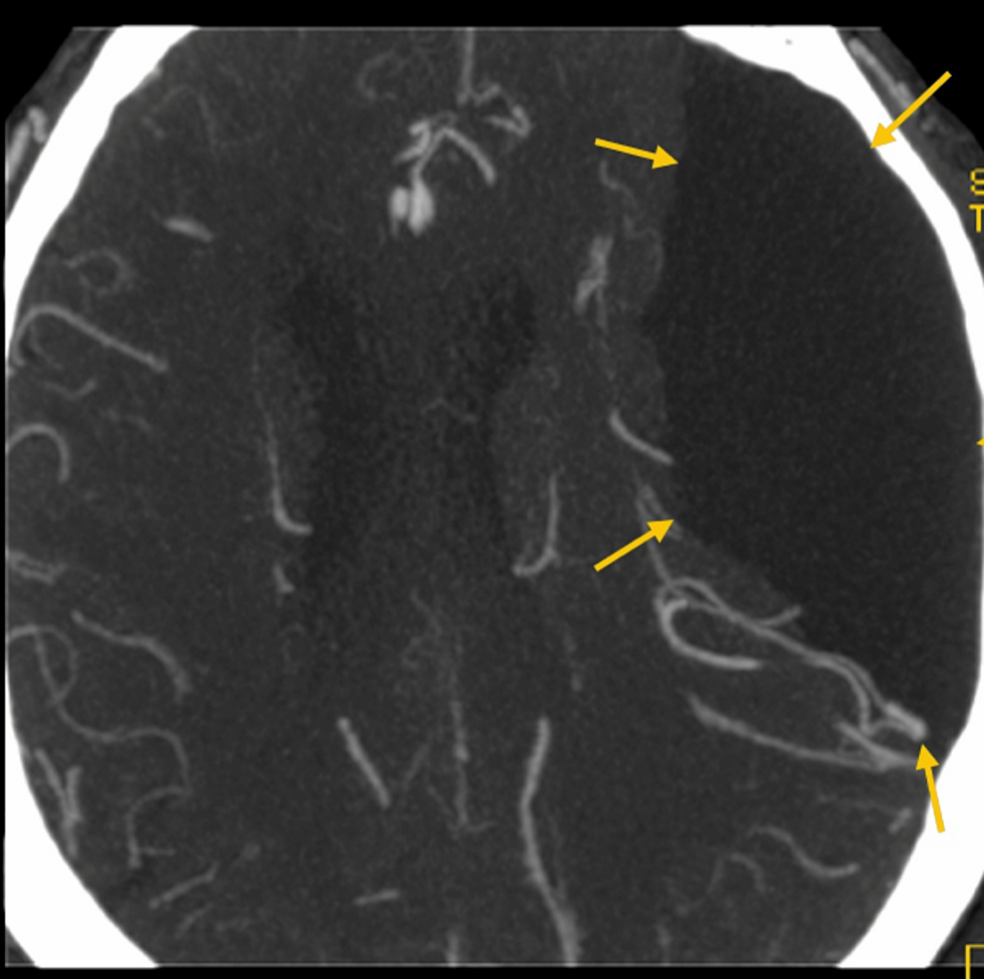
Brain computed tomography with angiography Brain computed tomography with angiography in axial view showing the large extra-axial fluid collection and its compressing effect on the brain parenchyma and vasculature.

The authors describe a case of a 66-year-old autonomous man with a known history of benign prostatic hypertrophy, who presented to the emergency department for the first time with sudden onset of dysarthria, left central facial palsy, and left hemihypesthesia involving the tongue. No other focal neurological deficits were detected. The patient was hemodynamically stable, with a blood pressure of 153/84 mmHg and heart rate of 80 bpm, with normal peripheral oxygen blood saturation (SpO2 = 97%), and was normoglycemic. Traumatic brain injury and possible toxic exposures were ruled out. Assuming a stoke, a National Institutes of Health Stroke Scale (NIHSS) score of 5 was calculated and the patient promptly underwent a brain CT scan, followed by a brain CT angiography (Figures 1, 2), which revealed a large left fronto-parieto-temporal arachnoid cyst, with approximately 9.5 x 5.1 cm of maximum diameters (anteroposterior and transversal, respectably), compressing the brain parenchyma and the ventricular system with a right deviation of the median structures by about 5 mm. During the five-hour stay in the emergency department, the patient had a complete spontaneous resolution of the initial symptoms. He declined admission to the ward for observation and further investigation, choosing to be discharged against medical advice. A few weeks later, the patient was re-admitted to the hospital with the same deficits described at the initial episode but this time with severe dysarthria and new onset of left hemiparesis scoring 10 on the NIHSS. The patient was admitted to the neurosurgery ward and submitted to an emergent cystoperitoneal shunt. The procedure underwent without complications with complete remission of the initial deficits. One year later, the patient remained asymptomatic with follow-up brain CT scans showing no signs of surgery-related complications or cyst relapse.

## Discussion

Arachnoid cysts are common and usually non-complicated conditions, mostly incidentally found in neuroimaging exams performed due to other reasons. Usually classified according to their location, they can be grouped in suprasellar, middle cranial fossa, interhemispheric, and quadrigeminal arachnoid cysts. Suprasellar cysts are usually proximal to the third ventricle, presenting with hydrocephalus symptoms. Interhemispheric arachnoid cysts are usually unilateral, midline, or parasagittal, away from ventricles, and usually not causing hydrocephalus [4]. Quadrigeminal cistern cysts are uncommon but usually compress the cerebral aqueduct at an early stage, presenting with hydrocephalus when symptomatic, and their treatment is imperative [5]. Arachnoid cysts located in the middle cranial fossa (approximately 50-65% of related cases) can be classified with the Galassi classification: type I cysts are small and usually asymptomatic, with anterior middle cranial fossa location; type II cysts are located superiorly along the Sylvian fissure displacing the temporal lobe; and type III cysts, like the case described, are exceptionally large, being able to take up the entire middle cranial fossa, displacing the temporal, parietal, and frontal lobes [6].

According to the literature, only approximately 5% of patients with arachnoid cysts have cyst-related neurological symptoms. Symptoms frequently develop with an insidious and intermittent presentation related to the cyst growth or, in some cases, more abruptly if the cyst growth rate is too fast. An abrupt presentation with a total recovery of symptoms within a couple of hours is rarely described in the literature, especially with such significant size cysts. The available therapeutic strategies have been revised by many experts, but a consensual approach is still not defined, particularly in the majority of patients with moderate and unspecific symptoms. Cystoperitoneal shunt insertion, craniotomy, endoscopic fenestration, and marsupialization of the cyst are usually the available strategies, proven to be usually effective. Endoscopic fenestration of suprasellar cysts is mostly accepted as the best modality of treatment in detriment of open procedures, which are related to higher morbidity [7]. Fenestration and excision are the most accepted procedures for interhemispheric cysts [4]. Relating to quadrigeminal cysts, the preferred approach depends on the specific location, but still, some authors report success rates as high as 90% with the endoscopic approach being suggested as the first line of treatment [8]. Middle cranial fossa arachnoid cysts usually rely on MRI to help decide between endoscopic, microsurgical fenestration, and shunting. Nevertheless, in recent years, neurosurgeons are increasingly turning to endoscopic techniques, when possible, with high success rates and safety [3]. Shunting is usually avoided due to the complications related to long-term shunt placement. The cyst location is still important in surgical decision-making. In this setting, it is consensually accepted that the need for treatment depends mostly upon the location and size of the cyst. If the cyst is small, not disturbing the surrounding tissue, and therefore asymptomatic, the treatment can be postponed; however, it is known that in these cases, the risk of intracranial bleeding after minor trauma is significant. If symptoms develop, untreated arachnoid cysts may lead to permanent severe neurological damage when the progressive expansion of the cyst or bleeding into the cyst injures the brain or spinal cord [9,10].

In cases like the one described, neurosurgery becomes mandatory, either by craniotomy resection of the cyst wall and communication with the basal cisterns or construction of cystoperitoneal drainage to prevent permanent compressive neurovascular damage. Recent data released by some authors support that the minimally invasive endoscopic approach to treat such cases has the potential for fewer complication rates, but it is still not consensual, and results are dependent on operator experience [2,11,12]. Nevertheless, in the presented case, the shunting strategy decision resulted from the lack of operator experience performing the endoscopic procedure.

## Conclusions

Although arachnoid cysts are relatively common findings in neuroimaging, symptomatic arachnoid cysts remain rare, probably due to their very slow development. Depending on cyst size and location, when surgery is indicated, many techniques are available. Although some location preferential treatment strategies may be considered, the lack of extensive studies, the rareness of cases needing intervention, and the operator experience-dependent outcome make the management of this medical condition still controversial, especially in small medical centers like ours. The presented case becomes especially interesting when we consider the onset timing of symptoms and the exuberance of the described cyst and its mass effect over the brain parenchyma. The total recovery within a couple of hours without any specific intervention is fascinating and reveals the amazing plasticity capabilities of the brain. Waiting for the development of severe symptoms before surgery is non-consensual due to the increased possible complications like bleeding or permanent brain damage. Therefore, the strategic approach should be discussed with the patient regarding the risks and benefits of any individual situation.
